# Ten novel psychrophilic Flavobacterium species from Tibetan Plateau glaciers define a cryospheric lineage with global cold-origin relatives

**DOI:** 10.1099/ijsem.0.007021

**Published:** 2026-01-13

**Authors:** Qing Liu, Lei-Lei Yang, Yu-Hua Xin

**Affiliations:** 1 State Key Laboratory of Microbial Diversity and Innovative Utilization, Institute of Microbiology, Chinese Academy of Sciences, Beijing 100101, PR China; 2 China General Microbiological Culture Collection Center (CGMCC), Institute of Microbiology, Chinese Academy of Sciences, Beijing 100101, PR China; 3 Beijing Key Laboratory of Genetic Element Biosourcing & Intelligent Design for Biomanufacturing, Beijing 100101, PR China

**Keywords:** cryospheric lineage, *Flavobacterium*, glacier, polar regions, psychrophilic

## Abstract

Twenty-three novel psychrophilic bacteria, represented by the type strains LB2P44^T^, LB2P6^T^, LB1P62^T^, LB3P6^T^, LS1P3^T^, XS2P39^T^, XS1P32^T^, ZB4P13^T^, ZS1P14^T^ and GT2P42^T^, were isolated from ice, cryoconite and meltwater of five Tibetan Plateau glaciers. All strains were Gram-stain-negative, aerobic, rod-shaped and psychrophilic, with optimal growth at 14–20 °C and pH 7.0. 16S rRNA gene sequence similarities to validly named *Flavobacterium* species ranged from 98.12% to 99.56%. Phylogenomic analysis of 81 concatenated core genes positioned the 23 strains (comprising the ten novel species) within a robust monophyletic clade — the ‘Cryospheric Lineage’ — together with 31 other psychrophilic type strains predominantly from glaciers, permafrost and polar regions. Average nucleotide identity (ANI) values of ≤94.4% and digital DNA–DNA hybridization (dDDH) values of ≤57.3% against closest relatives were below species thresholds (95–96% ANI, 70% dDDH). Major fatty acids were iso-C_15:0_ (8.1–16.8%), iso-C_15:0_ 3-OH (3-hydroxy, 5.2–16.0%) and summed feature 3 (C_16:1_* ω*7*c* and/or C_16:1_* ω*6*c*, 10.1–27.6%), with elevated branched-chain and unsaturated components typical of cold adaptation. Polyphasic taxonomic evidence supports the description of ten novel species within the genus *Flavobacterium: Flavobacterium amylolyticum* sp. nov. (LB2P44^T^=CGMCC 1.11256^T^=NBRC 114815^T^), *Flavobacterium glucosi* sp. nov. (LB2P6^T^=CGMCC 1.11263^T^=NBRC 114816^T^), *Flavobacterium esculini* sp. nov. (LB1P62^T^=CGMCC 1.11346^T^=NBRC 114817^T^), *Flavobacterium labens* sp. nov. (LB3P6^T^=CGMCC 1.11428^T^=NBRC 114818^T^), *Flavobacterium pasteuri* sp. nov. (LS1P3^T^=CGMCC 1.11474^T^=NBRC 114821^T^), *Flavobacterium rhamnosi* sp. nov. (XS2P39^T^=CGMCC 1.23204^T^=NBRC 115054^T^), *Flavobacterium frigidum* sp. nov. (XS1P32^T^=CGMCC 1.23370^T^=NBRC 115055^T^), *Flavobacterium glycogeni* sp. nov. (ZB4P13^T^=CGMCC 1.24050^T^=NBRC 115056^T^), *Flavobacterium kochi* sp. nov. (ZS1P14^T^=CGMCC 1.24093^T^=NBRC 114828^T^) and *Flavobacterium cryophilum* sp. nov. (GT2P42^T^=CGMCC 1.24821^T^=NBRC 114831^T^).

## Introduction

Glaciers, characterized by their extreme cold and oligotrophic conditions, host specialized bacterial communities uniquely adapted to these harsh ecosystems. The genus *Flavobacterium*, within the phylum *Bacteroidota*, class *Flavobacteriia*, order *Flavobacteriales* and family *Flavobacteriaceae*, comprises Gram-negative bacteria that typically form yellow-pigmented colonies [1]. As of September 2025, the genus includes 332 validly published species [2] (https://lpsn.dsmz.de/genus/flavobacterium), widely distributed across diverse environments, such as marine environments, glaciers, polar regions, lakes, rivers, sediments and soils [1]. This ecological versatility underscores the genetic and phenotypic diversity of *Flavobacterium*. Recent studies have provided insights into the temperature adaptation mechanisms of *Flavobacterium*. In glacier surface ecosystems, *Flavobacterium* ranks among the dominant bacterial groups [3], exhibiting pronounced psychrophilic traits. The cell membranes of psychrophilic *Flavobacterium* strains are enriched with branched-chain and unsaturated fatty acids, which enhance membrane fluidity at low temperatures [4]. Furthermore, these bacterial species possess cold-adaptation genes and specific amino acid substitutions that improve protein flexibility under cold conditions [5].

Global warming, one of the most significant climate shifts in human history, severely impacts cold environments such as glaciers and polar regions. Glacier retreat poses significant challenges to microbial communities, particularly indigenous psychrophilic bacteria. Some species, such as *Flavobacterium laiguense* [6], *Flavobacterium xylosi* [7] and *Flavobacterium restrictum* [8], are unable to survive at temperatures above 20 °C and face the risk of extinction. Consequently, investigating bacterial diversity and identifying novel species in glacial environments are critical for advancing fundamental science and addressing the urgent need to conserve microbial diversity in these vulnerable ecosystems.

In this study, we isolated 23 *Flavobacterium* strains from the surface ecosystems of five glaciers on the Qinghai–Tibet Plateau. Using a polyphasic taxonomic approach, we identified these strains as belonging to ten novel species, significantly expanding the known diversity of *Flavobacterium* in glacial habitats. Phylogenomic analysis revealed that these novel strains, along with 31 other type strains, form a distinct clade, predominantly composed of psychrophilic species originating from cryospheric environments. We propose the term ‘Cryospheric Lineage’ to describe this *Flavobacterium* clade, which is exclusively associated with glaciers, permafrost, polar regions and other cold environments. This concept enhances our understanding of *Flavobacterium* biogeography and provides a valuable model for studying the adaptive evolution of micro-organisms in cold environments.

## Isolation and ecology

Ice, cryoconite and meltwater samples were collected in October 2016 from five glaciers on the Qinghai–Tibet Plateau: Laigu Glacier (29.3087826 N, 96.8186951 E), Zepu Glacier (30.276556 N, 95.2508392 E), Zhuxi Glacier (30.045208 N, 95.5828705 E), Gawalong Glacier (29.7659264 N, 95.71035 E) and Renlongba Glacier (29.2615929 N, 96.9359436 E). After collection, the samples were stored in sterile bags, transported to the laboratory under low-temperature conditions, homogenized in sterile water and subjected to ten-fold serial dilutions. Aliquots (200 µl) from each dilution were spread onto peptone-yeast extract-glucose (PYG) and Reasoner’s 2A (R2A) agar plates, which were incubated at 14 °C for up to 30 days. More than 2,900 bacterial strains were picked, purified and classified into 137 genera. *Pseudomonas* (12.7%), *Flavobacterium* (11.4%) and *Cryobacterium* (8.1%) were the most abundant genera. In this study, 23 *Flavobacterium* strains (Table 1) were selected for polyphasic taxonomic analysis. These strains were preserved in 10% (v/v) glycerol suspensions and stored in liquid nitrogen for long-term conservation.

**Table 1.** The information on the 23 *Flavobacterium* strains in this study

Strain	CGMCC no.	NBRC no.	Proposed name	Source	Location
LB2P44^T^	1.11256	114815	*F. amylolyticum*	Ice	Laigu Glacier
LB2R40	1.11753		*F. amylolyticum*	Ice	Laigu Glacier
LB2P6^T^	1.11263	114816	*F. glucosi*	Ice	Laigu Glacier
LB2P74	1.11367		*F. glucosi*	Ice	Laigu Glacier
RSP29	1.24456		*F. glucosi*	Meltwater	Renlongba Glacier
LB1P62^T^	1.11346	114817	*F. esculini*	Ice	Laigu Glacier
LB1P71	1.11347		*F. esculini*	Ice	Laigu Glacier
GSP11	1.24171		*F. esculini*	Meltwater	Gawalong Glacier
LB3P6^T^	1.11428	114818	*F. labens*	Ice	Laigu Glacier
LB3P21	1.11431		*F. labens*	Ice	Laigu Glacier
LB3R33	1.11556		*F. labens*	Ice	Laigu Glacier
LS2R12	1.11773		*F. labens*	Meltwater	Laigu Glacier
XS1P27	1.23177		*F. labens*	Meltwater	Zhuxi Glacier
LS1P3^T^	1.11474	114821	*F. pasteuri*	Meltwater	Laigu Glacier
XS2P39^T^	1.23204	115054	*F. rhamnosi*	Meltwater	Zhuxi Glacier
XS1P32^T^	1.23370	115055	*F. frigidum*	Meltwater	Zhuxi Glacier
ZT3P35	1.23439		*F. frigidum*	Cryoconite	Zepu Glacier
GSP14	1.24980		*F. frigidum*	Meltwater	Gawalong Glacier
XS2P14	1.23355		*F. frigidum*	Meltwater	Zhuxi Glacier
ZB4P13^T^	1.24050	115056	*F. glycogeni*	Ice	Zepu Glacier
ZS1P14^T^	1.24093	114828	*F. kochi*	Meltwater	Zepu Glacier
GT2P42^T^	1.24821	114831	*F. cryophilum*	Cryoconite	Gawalong Glacier
GT2N3	1.24859		*F. cryophilum*	Cryoconite	Gawalong Glacier

## 16S rRNA phylogeny

Genomic DNA was extracted using the TaKaRa MiniBEST Bacteria Genomic DNA Extraction Kit according to the manufacturer’s instructions. The 16S rRNA gene was amplified with universal primers 27F and 1492R [9] and sequenced by SinoGenoMax (China) using the Sanger method. Sequences were compared against the EzBioCloud database for identification [10]. Multiple sequence alignments were performed using MAFFT version 7.520 with default settings [11]. Phylogenetic trees were constructed using mega software version 12 [12], employing neighbour-joining (NJ) and maximum-likelihood (ML) methods, with 1,000 bootstrap replicates to assess tree robustness. Genetic distances for NJ analysis were calculated using Kimura’s two-parameter model.

**Fig. 1.**
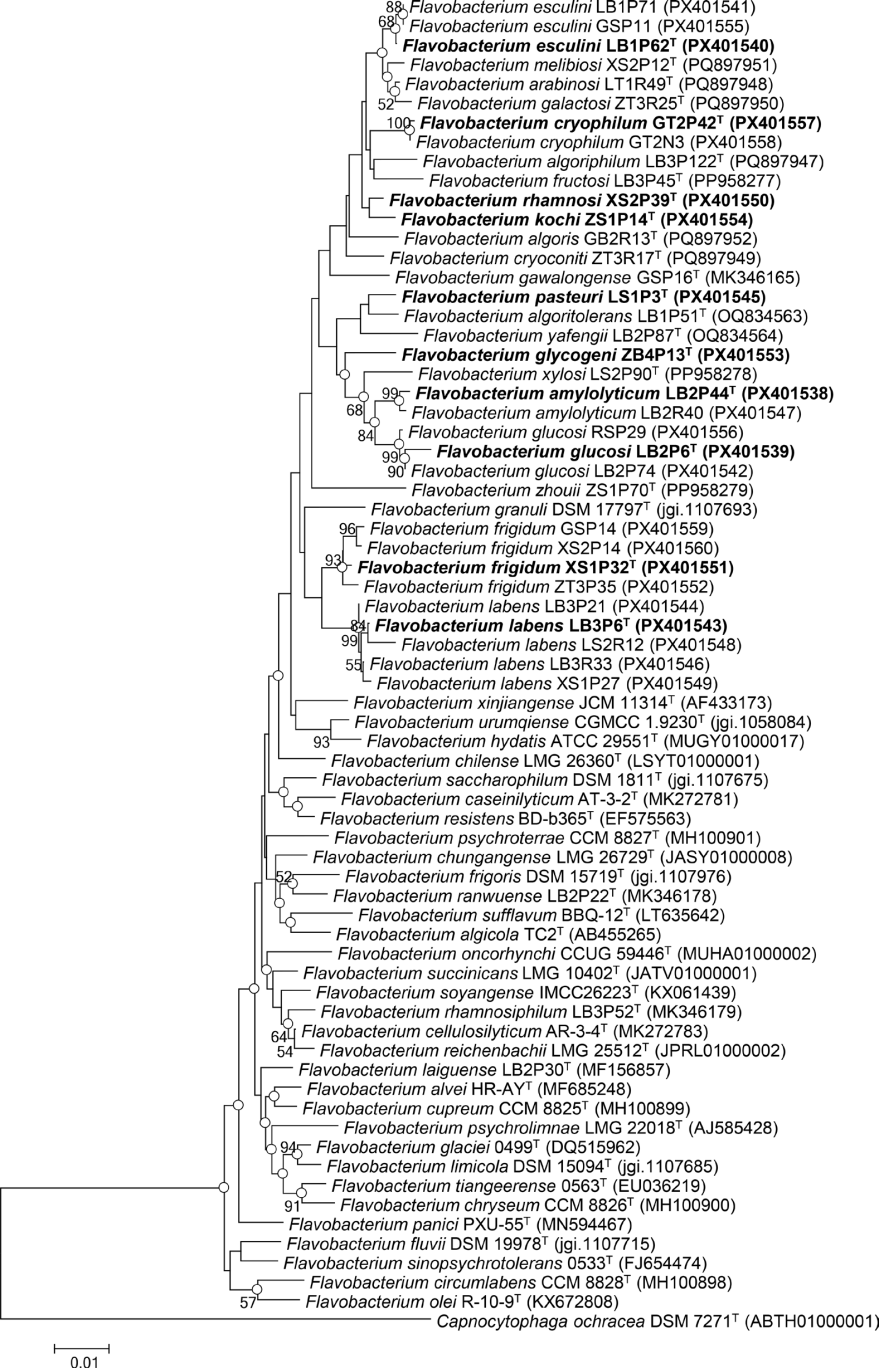
Phylogenetic tree of 23 strains and closely related strains based on 16S rRNA gene sequence comparisons using the NJ method. GenBank accession numbers for the 16S rRNA gene sequences are given in parentheses. Open circles indicate corresponding branches that were recovered in both the ML and NJ trees. Bootstrap values (>50%), based on 1,000 replicates, are shown at the branch nodes. Bar, 0.01 substitutions per nucleotide position.

The 16S rRNA gene sequence comparisons revealed that these 23 strains belong to the genus *Flavobacterium*, showing 98.12–99.56% similarity with *Flavobacterium sinopsychrotolerans* 0533^T^ (98.69–98.77%), *Flavobacterium urumqiense* Sr25^T^ (98.12–98.76%), *Flavobacterium galactosi* ZT3R25^T^ (99.34–99.56%), *Flavobacterium glaciei* 0499^T^ (98.26–99.06%), *Flavobacterium algoritolerans* LB1P51^T^ (98.97%), *Flavobacterium arabinosi* LT1R49^T^ (99.48%) and *Flavobacterium melibiosi* XS2P12^T^ (98.48–98.56%) (Table S1, available in the online Supplementary Material). The phylogenetic analysis of 16S rRNA gene sequences was conducted using the NJ method, which confirmed their placement within the genus *Flavobacterium* (Fig. 1). These strains formed a distinct lineage, primarily comprising strains from glaciers, such as *Flavobacterium algoriphilum* LB3P122^T^ [13], *F. algoritolerans* LB1P51^T^ [14], *Flavobacterium algoris* GB2R13^T^ [7], *F. xylosi* LS2P90^T^ [7], *F. urumqiense* CGMCC 1.9230^T^ [15] and *Flavobacterium yafengii* LB2P87^T^ [14]. Although NJ and ML trees showed similar topologies, most nodes had bootstrap values below 50%, indicating an unstable 16S rRNA phylogeny.

## Genome features

Whole-genome sequencing was conducted on the Illumina HiSeq 4000 platform (Illumina, San Diego, CA, USA) with 150 bp paired-end reads. *De novo* assembly was performed using SPAdes version 3.15 [16]. Genome completeness and contamination were evaluated with CheckM2 version 1.0.2 [17], and assembly quality was assessed using QUAST version 5.0.2 [18]. A phylogenomic tree was constructed with IQ-TREE 2 [19] based on a concatenated dataset of 81 single-copy core genes identified by the UBCG2 pipeline [20]. Alignments were generated using MAFFT version 7.520 [11], and the tree was evaluated with 1,000 bootstrap replicates under the GTR+F+R9 model. Average nucleotide identity (ANI) was calculated using FastANI version 1.33 [21], and digital DNA–DNA hybridization (dDDH) values were determined via the Type (Strain) Genome Server (TYGS) [22]. Gene prediction and annotation were performed with Prokka version 1.14 [23].

**Fig. 2.**
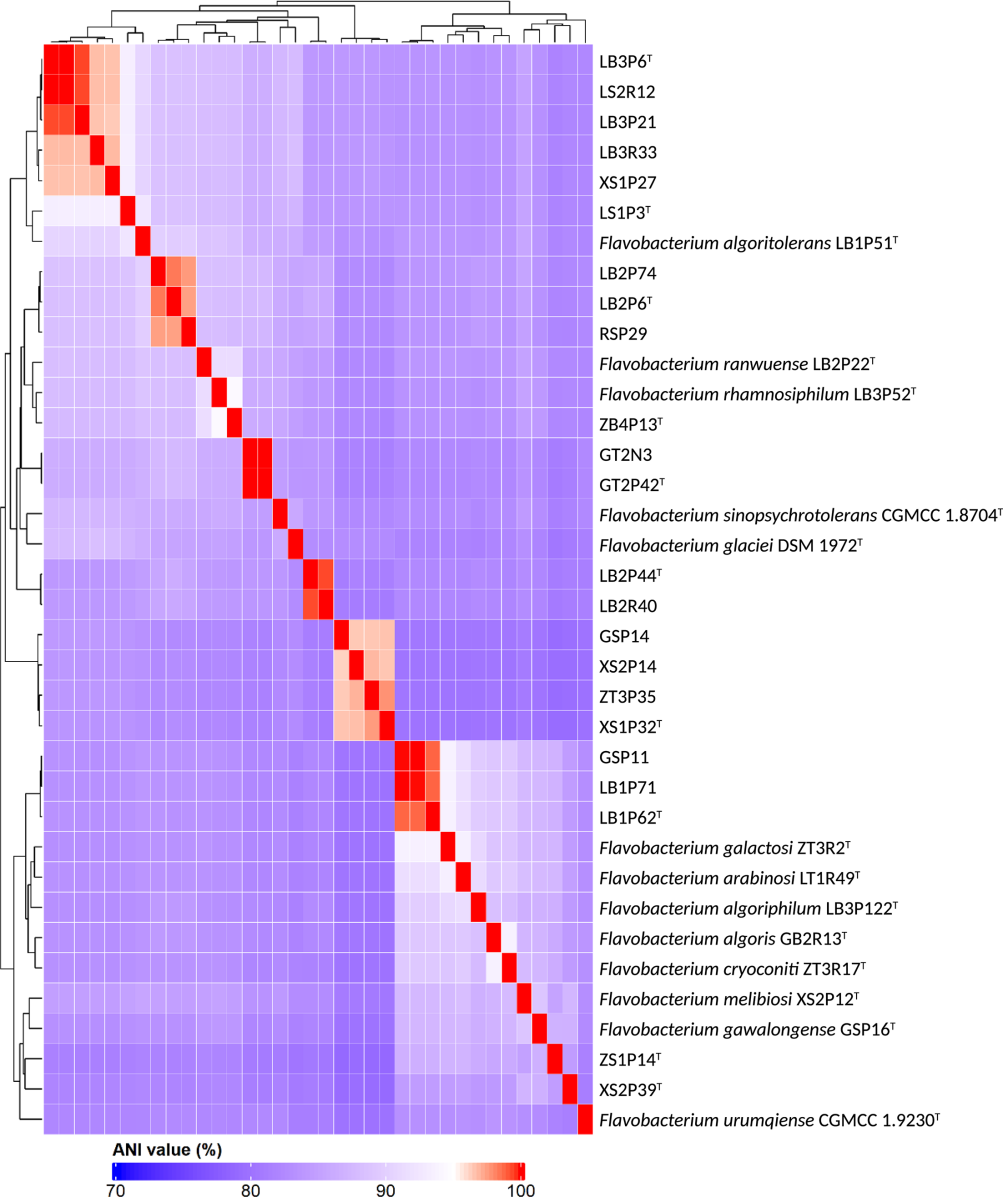
Heatmap of pairwise ANI values of the 23 strains and their closely related taxa.

Genomic sequencing of the 23 *Flavobacterium* strains produced high-quality assemblies, with genome completeness ranging from 99.94% to 100% and contamination from 0.01% to 0.98% (Table S2). For the ten type strains, genome sizes varied from 3.30 Mb (LB2P44^T^) to 5.23 Mb (ZS1P14^T^), with contig numbers between 26 and 236 and G+C contents from 33.73 to 35.29 mol%. Their coding sequences (CDSs) ranged from 2,874 to 4,389, accompanied by 10–49 tRNAs, 5–9 rRNAs, 77–121 miscellaneous RNAs (misc RNAs) and one transfer-messenger RNA (tmRNA) per strain. The 13 non-type strains exhibited similar ranges in genomic features: CDSs numbered 2,915–3,497, tRNAs 43–50, rRNAs 5–9, misc RNAs 74–158 and each also contained one tmRNA (Table S3). To confirm their taxonomic status, ANI (79.71–94.39%) and dDDH (19.5–57.3%) values were calculated between their closest relatives. The highest ANI value (94.39%) was observed between ZB4P13^T^ and *Flavobacterium rhamnosiphilum* LB3P52^T^, whereas all other isolate–type strain pairs showed ANI values <93.57%. The highest dDDH value (57.3%) occurred between ZB4P13^T^ and *F. rhamnosiphilum* CGMCC 1.11446^T^, followed by 51.7% between LB1P62^T^ and *F. galactosi* CGMCC 1.11711^T^. Consistent with these genome-relatedness indices, the ANI heatmap (Fig. 2) and the genome distance phylogeny inferred using TYGS (Fig. S1) confirmed the 23 strains as representing ten novel *Flavobacterium* species [24,25]. Four novel species were represented by single strains (LS1P3^T^, ZB4P13^T^, ZS1P14^T^, XS2P39^T^), whereas the remaining six were represented by 2–5 strains, with ANI values ≥96% and dDDH values ≥70%.

**Fig. 3.**
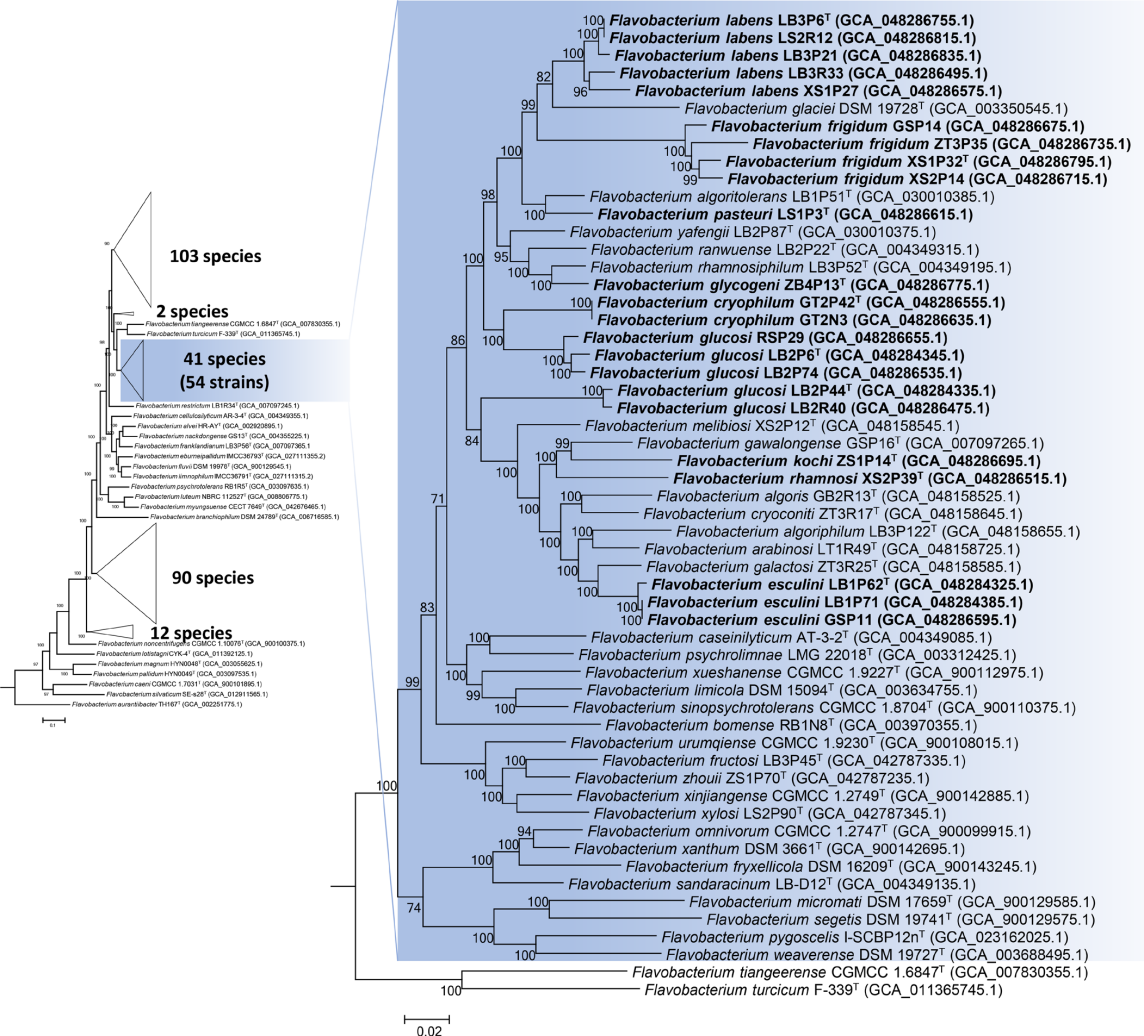
Phylogenomic tree, constructed based on 81 core genes, delineates the evolutionary relationships among the 23 novel strains and their closely related type strains within the genus *Flavobacterium*. Strains highlighted with a blue background belong to the ‘Cryospheric Lineage’. Genomic sequence accession numbers are given in parentheses. Bootstrap values (>70%), based on 1,000 replicates, are shown at the branch nodes. Bar, 0.1 substitutions per site (left) and 0.02 substitutions per site (right).

To elucidate the phylogenetic relationships of these novel species, a robust phylogenomic tree was constructed using genomic sequences of 259 known *Flavobacterium* species, along with the 23 novel strains (Fig. 3). The 23 strains formed ten distinct branches separate from other named species, supporting the ANI and dDDH results (Fig. 3). Notably, these strains, along with 31 other type strains, formed a distinct lineage with 100% bootstrap support. Except for *Flavobacterium limicola* DSM 15094^T^, a psychrophilic strain isolated from freshwater [26], all members of this lineage originated from cryospheric environments, such as *Flavobacterium caseinilyticum* AT-3-2^T^ from the Arctic [27], *Flavobacterium psychrolimnae* LMG 22018^T^ from the Antarctic [28] and 44 strains from glaciers in the Tibetan Plateau or in Xinjiang Province, China. We propose the term ‘Cryospheric Lineage’ to describe this *Flavobacterium* clade, encompassing species isolated from glaciers, permafrost, the Arctic and Antarctic regions and other cold environments. This lineage significantly enhances our understanding of *Flavobacterium* diversity in cold environments and provides a framework for studying microbial biogeography and adaptive evolution.

## Physiology and chemotaxonomy

Colony morphology was examined on PYG agar plates, and Gram staining was performed using standard protocols. Cell morphology was observed via transmission electron microscopy (JEM-1400, JEOL Ltd., Tokyo, Japan). Cytochrome oxidase activity was tested with 1% (w/v) tetramethyl-*p*-phenylenediamine, and catalase activity was assessed by bubble formation in 3% (v/v) H_2_O_2_. Gliding motility was assessed using phase-contrast microscopy after growth on 1/4-strength R2A agar [29]. Growth was tested in PYG broth over a temperature range of 0–30 °C, a pH range of 4.0–10.0 (in 1-unit intervals, adjusted with 0.2 M Na_2_HPO_4_/NaH_2_PO_4_ for pH 5–8 and 0.2 M Na_2_CO_3_/NaHCO_3_ for pH 9–10) and NaCl concentrations of 0–4% (w/v) in 0.5% increments. Flexirubin-type pigments were detected using 20% (w/v) KOH. Casein hydrolysis was assayed with 2.5% (w/v) skimmed milk according to the method of Smibert and Krieg [30]. Starch and Tween 80 hydrolysis were determined on PYG agar as the basal medium. Carbon source utilization was examined in a basal medium [containing 0.2% (NH_4_)2SO_4_, 0.05% NaH_2_PO_4_·H_2_O, 0.05% K_2_HPO_4_, 0.02% MgSO_4_·7H_2_O and 0.01% CaCl_2_·2H_2_O] supplemented with 1% (w/v) of various carbon compounds listed in Table S4. Additional biochemical characteristics were determined using API 20NE, API 20E and API ZYM strips (bioMérieux, Marcy l'Étoile, France). Cell suspensions were prepared by harvesting fresh colonies from PYG agar and resuspending them in sterile water to obtain a homogeneous inoculum, which was then used to inoculate the API systems according to the manufacturer’s instructions. API 20NE and API 20E strips were incubated at 14 °C and read after 7 days, whereas API ZYM strips were incubated at 14 °C, and the reactions were recorded after 4–8 h. For fatty acid analysis, cells were harvested during the late exponential phase in PYG medium at 14 °C. Fatty acids were extracted and detected using gas chromatography (Agilent 6890 N; Agilent Technologies, Santa Clara, CA, USA) and analysed using the MIDI 6.0 system (MIDI Inc., Newark, DE, USA), with profiles compared against the Trypticase Soy Broth Agar version 6.0 (TSBA6) database [31].

Cells of these strains are rod-shaped (Fig. S2), yellow- or orange-pigmented (Fig. S3), catalase-positive and non-flagellated, exhibiting typical *Flavobacterium* characteristics. Comprehensive phenotypic analysis (Table 2) revealed clear interspecies differences in multiple physiological and biochemical traits, which are valuable for species delineation. All strains exhibited maximum growth temperatures between 21 and 27 °C and were unable to grow at 30 °C, consistent with their obligate psychrophilic nature and origin from glacial environments. Several traits displayed stable differentiation patterns across species, providing taxonomic value (Table 2). For instance, flexirubin-type pigments were detected only in strains ZB4P13^T^ and ZS1P14^T^, whereas gliding motility was observed in strains LB1P62^T^, LB1P71, GSP11, LB3P6^T^, LB3P21, LB3R33, LS2R12, XS1P27, LS1P3^T^ and XS2P39^T^, but absent in the remaining 13 strains. The strains also exhibited diverse enzymatic activities and substrate utilization profiles. All species produced alkaline phosphatase, esterase lipase (C8), lipase (C14), leucine arylamidase, valine arylamidase and cystine arylamidase, and could utilize d-glucose, d-galactose, maltose, lactose, d-mannose, glycogen and d-trehalose. However, traits such as the Voges–Proskauer test, hydrolysis of starch, casein, aesculin and gelatin, as well as certain enzyme activities and carbon source utilization, varied among species, serving as distinguishing characteristics. Notably, some carbon source utilization traits showed intraspecific variation (marked as +/− in Tables 2 and S4). For example, melibiose utilization varied among strains LB2P6^T^, LB2P74 and RSP29; melibiose, D-turanose and d-fructose utilization varied among strains XS1P32^T^, ZT3P35, GSP14 and XS2P14; l-rhamnose utilization varied among strains LB2P44^T^ and LB2R40; and d-fructose utilization varied among strains GT2P42^T^ and GT2N3. This intraspecific variation reflects the genetic plasticity of *Flavobacterium* populations, likely an adaptation to micro-niche differences in their natural environments. While these variable traits are not absolute markers for species delineation, they provide valuable insights into phenotypic diversity within species.

**Table 2.** Phenotypic characteristics that differentiate the ten novel species

Characteristic	1	2	3	4	5	6	7	8	9	10	11	12	13
Maximum growth temperature (°C)	23	25	25	25	27	25	25	25	25	22	25	25	21
Flexirubin-type pigments	−	−	−	−	−	−	−	−	−	−	+	+	−
Gliding motility	−	−	+	−	+	−	+	−	+	−	+	−	−
Voges–Proskauer test	−	+	+	+	+	−	+	+	+	−	+	+	+
**Hydrolysis of:**													
Starch	+	+	−	−	−	+	−	−	−	−	+	−	−
Casein	+	−	+	−	−	+	−	+	−	−	−	−	+
Aesculin	+	+	+	+	+	+	+	+	+	−	+	+	+
Gelatin	+	+	+	+	+	−	+	+	+	+	+	+	−
**Enzyme activity:**													
Arginine dihydrolase	−	+	−	+	−	+	−	+	−	−	−	−	−
Esterase (C4)	+	+	+	+	+	−	+	+	−	+	+	+	+
Trypsin	+	+	−	−	+	−	+	+	−	+	+	−	−
α-Chymotrypsin	+	−	−	−	+	−	+	−	+	+	+	−	+
α-Galactosidase	−	+	+	−	−	−	−	−	+	−	+	+	−
β-Galactosidase	+	+	+	+	+	−	+	+	+	−	+	+	+
β-Glucosidase	−	+	+	+	+	+	+	+	+	−	−	+	+
*N*-Acetyl-β-glucosaminidase	+	+	+	+	+	+	+	+	+	−	+	+	+
**Utilization of:**													
Melibiose	+	+/-	+	+	+	−	+	+	+	+/-	+	+	−
d-Xylose	−	−	+	+	−	−	−	−	−	−	−	+	−
d-Turanose	+	+	+	+	+	−	+	+	+	+/-	−	+	+
d-Fructose	+	+	+	+	+	−	+	+	+	+/-	−	+	+/-
l-Rhamnose	+/-	−	+	+	−	+	−	−	+	−	−	−	−
l-Proline	+	+	+	+	+	+	−	+	+	+	+	+	+
d-Raffinose	+	+	+	+	+	+	+	+	+	+	−	+	+
Sucrose	+	+	+	+	+	+	+	+	+	+	−	+	+
Cellobiose	+	+	+	+	+	+	+	+	+	+	−	+	+
l-Arabinose	+	+	+	+	+	−	+	+	+	+	−	+	+

1, LB2P44T, LB2R40; 2, LB2P6T, LB2P74, RSP29; 3, LB1P62T, LB1P71, GSP11; 4, *F. galactosi* ZT3R25T; 5, LB3P6T, LB3P21, LB3R33, LS2R12, XS1P27; 6, *F. glaciei* CGMCC 1.5380T; 7, LS1P3T; 8, *F. algoritolerans* LB1P51T; 9, XS2P39T; 10, XS1P32T, ZT3P35, GSP14, XS2P14; 11, ZB4P13T; 12, ZS1P14T; 13, GT2P42T, GT2N3. +, Positive; –, negative; +/-, variable.

Fatty acid analysis of the type strains of the ten novel species (Table S5) revealed profiles consistent with known *Flavobacterium* species [1]. The major fatty acids included iso-C_15:0_ (8.1–16.8%), iso-C_15:0_ 3-OH (3-hydroxy, 5.2–16.0%) and summed feature 3 (C_16:1_* ω*7*c* and/or C_16:1_* ω*6*c*, 10.1–27.6%). Relative abundances of major fatty acids varied among strains. For example, anteiso-C_15:0_ was low in LB2P44^T^ (3.1%) but high in XS2P39^T^ (14.3%). Summed feature 3 showed significant variation, peaking in LS1P3T (27.61%) but being lower in LB2P44^T^, LB2P6^T^, XS1P32^T^ and ZB4P13^T^ (~10–12%). Additionally, the presence or absence of specific fatty acids, such as iso-C_12:0_ (detected only in LB2P44^T^ and LB2P6^T^), facilitated species differentiation. Importantly, the fatty acid profiles of these strains exhibited typical psychrophilic adaptations, with high levels of branched-chain fatty acids (e.g. iso-C_15:0_, anteiso-C_15:0_) and unsaturated fatty acids (e.g. C_15:1_* ω*6*c*, C_17:1_* ω*6c, summed feature 3). This composition enhances membrane fluidity at low temperatures, representing a key physiological strategy for microbial adaptation to cold environments [5].

Based on integrated genomic, phylogenetic and physiological analyses, the 23 strains isolated from the Tibetan Plateau glaciers were confirmed to represent ten novel species within the genus *Flavobacterium*, significantly advancing our understanding of microbial diversity and adaptation in cryospheric ecosystems. Phylogenomic evidence further supports their placement within a distinct ‘Cryospheric Lineage’, highlighting their ecological specialization. The following novel species are proposed (Table 1), with their respective type strains and associated strains:

1. ***Flavobacterium amylolyticum* sp. nov**. (strains LB2P44^T^, LB2R40; type strain LB2P44^T^=CGMCC 1.11256^T^=NBRC 114815^T^)
2. ***Flavobacterium glucosi* sp. nov**. (strains LB2P6^T^, LB2P74, RSP29; type strain LB2P6^T^=CGMCC 1.11263^T^=NBRC 114816^T^)
3. ***Flavobacterium esculini* sp. nov**. (strains LB1P62^T^, LB1P71, GSP11; type strain LB1P62^T^=CGMCC 1.11346^T^=NBRC 114817^T^)
4. ***Flavobacterium labens* sp. nov**. (strains LB3P6^T^, LB3P21, LB3R33, LS2R12, XS1P27; type strain LB3P6^T^=CGMCC 1.11428^T^=NBRC 114818^T^)
5. ***Flavobacterium pasteuri* sp. nov**. (type strain LS1P3^T^=CGMCC 1.11474^T^=NBRC 114821^T^)
6. ***Flavobacterium rhamnosi* sp. nov**. (type strain XS2P39^T^=CGMCC 1.23204^T^=NBRC 115054^T^)
7. ***Flavobacterium frigidum* sp. nov**. (strains XS1P32^T^, ZT3P35, GSP14, XS2P14; type strain XS1P32^T^=CGMCC 1.23370^T^=NBRC 115055^T^)
8. ***Flavobacterium glycogeni* sp. nov**. (type strain ZB4P13^T^=CGMCC 1.24050^T^=NBRC 115056^T^)
9. ***Flavobacterium kochi* sp. nov**. (type strain ZS1P14^T^=CGMCC 1.24093^T^=NBRC 114828^T^)
10. ***Flavobacterium cryophilum* sp. nov**. (strains GT2P42^T^, GT2N3; type strain GT2P42^T^=CGMCC 1.24821^T^=NBRC 114831^T^).

## Protologues

### Description of *Flavobacterium amylolyticum* sp. nov.

*Flavobacterium amylolyticum* (a.my.lo.ly’ti.cum. Gr. neut. n. *amylon*, starch; N.L. masc. adj. *lyticus*, able to loosen, able to dissolve; from Gr. masc. adj. *lytikos,* able to loosen, dissolving; N.L. neut. adj. *amylolyticum*, starch-dissolving, referring to the property of being able to hydrolyse starch).

Cells are Gram-stain-negative, rod-shaped, non-gliding and devoid of flagella, measuring 0.6–0.8×1.8–4.5 µm. Colonies are circular, convex and yellow on PYG plates at 14 °C. Growth occurs at temperatures between 0 and 23 °C (optimum 14–20 °C), at pH 6.0–9.0 (optimum pH 7.0) and in the presence of 0–1.0% (w/v) NaCl. Flexirubin-type pigments are absent. Positive for catalase and negative for oxidase. Cells hydrolyse starch, casein, aesculin and gelatin, but do not hydrolyse Tween 80. Indole and H_2_S are not formed. Positive for alkaline phosphatase, esterase (C4), esterase lipase (C8), lipase (C14), leucine arylamidase, valine arylamidase, cystine arylamidase, trypsin, α-chymotrypsin, acid phosphatase, naphthol-AS-BI-phosphohydrolase, β-galactosidase, α-glucosidase and *N*-acetyl-β-glucosaminidase. Negative for the Voges–Proskauer test, reduction of nitrates to nitrites, fermentation of glucose, urease, arginine dihydrolase, lysine decarboxylase, ornithine decarboxylase, tryptophan deaminase, α-galactosidase, β-glucuronidase, β-glucosidase, α-mannosidase and α-fucosidase. Utilize the following carbohydrates as the sole carbon source: d-glucose, d-galactose, melibiose, maltose, lactose, d-mannose, d-turanose, d-fructose, glycogen, l-proline, d-raffinose, sucrose, d-trehalose, cellobiose and l-arabinose. The utilization of l-rhamnose is variable. Under the tested conditions, it does not utilize the following carbohydrates: d-mannitol, d-xylose, propionate, tartrate, l-sorbose, citrate, myo-inositol, succinate and d-ribose. The major fatty acids are iso-C_15:0_ and summed feature 3 (C_16:1_* ω*7*c* and/or C_16:1_* *ω*6c*). The genomic DNA G+C content of the type strain is 33.9 mol%.

The type strain LB2P44^T^ (=CGMCC 1.11256^T^=NBRC 114815^T^) was isolated from an ice sample collected from the Laigu Glacier on the Tibetan Plateau, PR China. The National Center for Biotechnology Information (NCBI) accession numbers for the 16S rRNA gene and genome sequences are PX401538 and JBLVPG000000000, respectively.

## Description of *Flavobacterium glucosi* sp. nov.

*Flavobacterium glucosi* (glu.co’si. N.L. gen. n. *glucosi*, of glucose).

Cells are Gram-stain-negative, rod-shaped, non-gliding and devoid of flagella, measuring 0.6–0.7×1.9–6.0 µm. Colonies are circular, convex and yellow on PYG plates at 14 °C. Growth occurs at temperatures between 0 and 25 °C (optimum 14–20 °C), at pH 6.0–8.0 (optimum pH 7.0) and in the presence of 0–1.0% (w/v) NaCl. Flexirubin-type pigments are absent. Positive for catalase and negative for oxidase. Cells hydrolyse starch, aesculin and gelatin, but do not hydrolyse casein or Tween 80. Indole and H_2_S are not formed. Positive for the Voges–Proskauer test, arginine dihydrolase, alkaline phosphatase, esterase (C4), esterase lipase (C8), lipase (C14), leucine arylamidase, valine arylamidase, cystine arylamidase, trypsin, acid phosphatase, naphthol-AS-BI-phosphohydrolase, α-galactosidase, β-galactosidase, α-glucosidase, β-glucosidase and *N*-acetyl-β-glucosaminidase. Negative for reduction of nitrates to nitrites, fermentation of glucose, urease, lysine decarboxylase, ornithine decarboxylase, tryptophan deaminase, α-chymotrypsin, β-glucuronidase, α-mannosidase and α-fucosidase. Utilize the following carbohydrates as the sole carbon source: d-glucose, d-galactose, maltose, lactose, d-mannose, d-turanose, d-fructose, glycogen, l-proline, d-raffinose, sucrose, d-trehalose, cellobiose and l-arabinose. The utilization of melibiose is variable. Under the tested conditions, it does not utilize the following carbohydrates: d-mannitol, d-xylose, l-rhamnose, propionate, tartrate, l-sorbose, citrate, myo-inositol, succinate and d-ribose. The major fatty acids are iso-C_15:0_, summed feature 3 (C_16:1_* ω*7*c* and/or C_16:1_* *ω*6c*) and iso-C_15:0_ 3-OH. The genomic DNA G+C content of the type strain is 34.3 mol%.

The type strain LB2P6^T^ (=CGMCC 1.11263^T^=NBRC 114816^T^) was isolated from an ice sample collected from the Laigu Glacier on the Tibetan Plateau, PR China. The NCBI accession numbers for the 16S rRNA gene and genome sequences are PX401539 and JBLVPH000000000, respectively.

## Description of *Flavobacterium esculini* sp. nov.

*Flavobacterium esculini* (es.cu.li’ni. N.L. gen. n. *esculini*, of aesculin).

Cells are Gram-stain-negative, rod-shaped, exhibiting gliding motility and devoid of flagella, measuring 0.7–0.8×1.8–3.3 µm. Colonies are circular, convex and yellow on PYG plates at 14 °C. Growth occurs at temperatures between 0 and 25 °C (optimum 14–20 °C), at pH 6.0–8.0 (optimum pH 7.0) and in the presence of 0–0.5% (w/v) NaCl. Flexirubin-type pigments are absent. Positive for catalase and oxidase. Cells hydrolyse casein, aesculin and gelatin, but do not hydrolyse starch or Tween 80. Indole and H_2_S are not formed. Positive for the Voges–Proskauer test, alkaline phosphatase, esterase (C4), esterase lipase (C8), lipase (C14), leucine arylamidase, valine arylamidase, cystine arylamidase, acid phosphatase, naphthol-AS-BI-phosphohydrolase, α-galactosidase, β-galactosidase, α-glucosidase, β-glucosidase and *N*-acetyl-β-glucosaminidase. Negative for reduction of nitrates to nitrites, fermentation of glucose, urease, arginine dihydrolase, lysine decarboxylase, ornithine decarboxylase, tryptophan deaminase, trypsin, α-chymotrypsin, β-glucuronidase, α-mannosidase and α-fucosidase. Utilize the following carbohydrates as the sole carbon source: d-glucose, d-galactose, melibiose, maltose, lactose, d-mannose, d-xylose, d-turanose, d-fructose, glycogen, l-rhamnose, l-proline, d-raffinose, sucrose, d-trehalose, cellobiose and l-arabinose. Under the tested conditions, it does not utilize the following carbohydrates: d-mannitol, propionate, tartrate, l-sorbose, citrate, myo-inositol, succinate and d-ribose. The major fatty acids are summed feature 3 (C_16:1_* ω*7*c* and/or C_16:1_* *ω*6c*), anteiso-C_15:0_ and iso-C_15:0_. The genomic DNA G+C content of the type strain is 33.8 mol%.

The type strain LB1P62^T^ (=CGMCC 1.11346^T^=NBRC 114817^T^) was isolated from an ice sample collected from the Laigu Glacier on the Tibetan Plateau, PR China. The NCBI accession numbers for the 16S rRNA gene and genome sequences are PX401540 and JBLVPI000000000, respectively.

## Description of *Flavobacterium labens* sp. nov.

*Flavobacterium labens* (la’bens. L. neut. part. adj. *labens*, gliding, referring to the gliding motility).

Cells are Gram-stain-negative, rod-shaped, exhibiting gliding motility and devoid of flagella, measuring 0.4–0.5×2.0–6.3 µm. Colonies are circular, convex and yellow on PYG plates at 14 °C. Growth occurs at temperatures between 0 and 27 °C (optimum 14–20 °C), at pH 6.0–9.0 (optimum pH 7.0) and in the presence of 0–1.0% (w/v) NaCl. Flexirubin-type pigments are absent. Positive for catalase and oxidase. Cells hydrolyse aesculin and gelatin, but do not hydrolyse starch, casein or Tween 80. Indole and H_2_S are not formed. Positive for the Voges–Proskauer test, alkaline phosphatase, esterase (C4), esterase lipase (C8), lipase (C14), leucine arylamidase, valine arylamidase, cystine arylamidase, trypsin, α-chymotrypsin, acid phosphatase, naphthol-AS-BI-phosphohydrolase, β-galactosidase, α-glucosidase, β-glucosidase and *N*-acetyl-β-glucosaminidase. Negative for reduction of nitrates to nitrites, fermentation of glucose, urease, arginine dihydrolase, lysine decarboxylase, ornithine decarboxylase, tryptophan deaminase, α-galactosidase, β-glucuronidase, α-mannosidase and α-fucosidase. Utilize the following carbohydrates as the sole carbon source: d-glucose, d-galactose, melibiose, maltose, lactose, d-mannose, d-turanose, d-fructose, glycogen, l-proline, d-raffinose, sucrose, d-trehalose, cellobiose and l-arabinose. Under the tested conditions, it does not utilize the following carbohydrates: d-mannitol, d-xylose, l-rhamnose, propionate, tartrate, l-sorbose, citrate, myo-inositol, succinate and d-ribose. The major fatty acids are summed feature 3 (C_16:1_* ω*7*c* and/or C_16:1_* *ω*6c*), C_17:1_* *ω*6c* and iso-C_15:0_. The genomic DNA G+C content of the type strain is 33.8 mol%.

The type strain LB3P6^T^ (=CGMCC 1.11428^T^=NBRC 114818^T^) was isolated from an ice sample collected from the Laigu Glacier on the Tibetan Plateau, PR China. The NCBI accession numbers for the 16S rRNA gene and genome sequences are PX401543 and JBLVPL000000000, respectively.

## Description of *Flavobacterium pasteuri* sp. nov.

*Flavobacterium pasteuri* (pas.teu’ri. N.L. gen. masc. n. *pasteuri*, honouring the French microbiologist Louis Pasteur).

Cells are Gram-stain-negative, rod-shaped, exhibiting gliding motility and devoid of flagella, measuring 0.6–0.7×1.7–6.2 µm. Colonies are circular, convex and yellow on PYG plates at 14 °C. Growth occurs at temperatures between 0 and 25 °C (optimum 14–20 °C), at pH 6.0–8.0 (optimum pH 7.0) and in the presence of 0–1.0% (w/v) NaCl. Flexirubin-type pigments are absent. Positive for catalase and oxidase. Cells hydrolyse aesculin and gelatin, but do not hydrolyse starch, casein or Tween 80. Indole and H_2_S are not formed. Positive for the Voges–Proskauer test, alkaline phosphatase, esterase (C4), esterase lipase (C8), lipase (C14), leucine arylamidase, valine arylamidase, cystine arylamidase, trypsin, α-chymotrypsin, acid phosphatase, naphthol-AS-BI-phosphohydrolase, β-galactosidase, α-glucosidase, β-glucosidase and *N*-acetyl-β-glucosaminidase. Negative for reduction of nitrates to nitrites, fermentation of glucose, urease, arginine dihydrolase, lysine decarboxylase, ornithine decarboxylase, tryptophan deaminase, α-galactosidase, β-glucuronidase, α-mannosidase and α-fucosidase. Utilize the following carbohydrates as the sole carbon source: d-glucose, d-galactose, melibiose, maltose, lactose, d-mannose, d-turanose, d-fructose, glycogen, d-raffinose, sucrose, d-trehalose, cellobiose and l-arabinose. Under the tested conditions, it does not utilize the following carbohydrates: d-mannitol, d-xylose, l-rhamnose, propionate, tartrate, l-sorbose, citrate, myo-inositol, succinate, d-ribose and l-proline. The major fatty acids are summed feature 3 (C_16:1_* ω*7*c* and/or C_16:1_* *ω*6c*) and iso-C_15:0_ 3-OH. The genomic DNA G+C content of the type strain is 33.7 mol%.

The type strain LS1P3^T^ (=CGMCC 1.11474^T^=NBRC 114821^T^) was isolated from a meltwater sample collected from the Laigu Glacier on the Tibetan Plateau, PR China. The NCBI accession numbers for the 16S rRNA gene and genome sequences are PX401545 and JBLVPN000000000, respectively.

## Description of *Flavobacterium rhamnosi* sp. nov.

*Flavobacterium rhamnosi* (rham.no’si. N.L. gen. n. *rhamnosi*, pertaining to rhamnose).

Cells are Gram-stain-negative, rod-shaped, exhibiting gliding motility and devoid of flagella, measuring 0.6–0.8×2.1–3.1 µm. Colonies are circular, convex and yellow on PYG plates at 14 °C. Growth occurs at temperatures between 0 and 25 °C (optimum 14–20 °C), at pH 6.0–8.0 (optimum pH 7.0) and in the presence of 0–0.5% (w/v) NaCl. Flexirubin-type pigments are absent. Positive for catalase and oxidase. Cells hydrolyse aesculin and gelatin, but do not hydrolyse starch, casein or Tween 80. Indole and H_2_S are not formed. Positive for the Voges–Proskauer test, alkaline phosphatase, esterase lipase (C8), lipase (C14), leucine arylamidase, valine arylamidase, cystine arylamidase, α-chymotrypsin, acid phosphatase, naphthol-AS-BI-phosphohydrolase, α-galactosidase, β-galactosidase, α-glucosidase, β-glucosidase and *N*-acetyl-β-glucosaminidase. Negative for reduction of nitrates to nitrites, fermentation of glucose, urease, arginine dihydrolase, lysine decarboxylase, ornithine decarboxylase, tryptophan deaminase, esterase (C4), trypsin, β-glucuronidase, α-mannosidase and α-fucosidase. Utilize the following carbohydrates as the sole carbon source: d-glucose, d-galactose, melibiose, maltose, lactose, d-mannose, d-turanose, d-fructose, glycogen, l-rhamnose, l-proline, d-raffinose, sucrose, d-trehalose, cellobiose and l-arabinose. Under the tested conditions, it does not utilize the following carbohydrates: d-mannitol, d-xylose, propionate, tartrate, l-sorbose, citrate, myo-inositol, succinate and d-ribose. The major fatty acids are summed feature 3 (C_16:1_* ω*7*c* and/or C_16:1_* *ω*6c*), anteiso-C_15:0_ and iso-C_15:0_. The genomic DNA G+C content of the type strain is 34.7 mol%.

The type strain XS2P39^T^ (=CGMCC 1.23204^T^=NBRC 115054^T^) was isolated from a meltwater sample collected from the Zhuxi Glacier on the Tibetan Plateau, PR China. The NCBI accession numbers for the 16S rRNA gene and genome sequences are PX401550 and JBLVPS000000000, respectively.

## Description of *Flavobacterium frigidum* sp. nov.

*Flavobacterium frigidum* (fri’gi.dum. L. neut. adj. *frigidum*, cold).

Cells are Gram-stain-negative, rod-shaped, non-gliding and devoid of flagella, measuring 0.7–1.0×2.4–5.0 µm. Colonies are circular, convex and yellow on PYG plates at 14 °C. Growth occurs at temperatures between 0 and 22 °C (optimum 14–20 °C), at pH 7.0–8.0 (optimum pH 7.0) and in the presence of 0–0.5% (w/v) NaCl. Flexirubin-type pigments are absent. Positive for catalase and oxidase. Cells hydrolyse gelatin, but do not hydrolyse aesculin, starch, casein or Tween 80. Indole and H_2_S are not formed. Positive for the alkaline phosphatase, esterase (C4), esterase lipase (C8), lipase (C14), leucine arylamidase, valine arylamidase, cystine arylamidase, trypsin, α-chymotrypsin, acid phosphatase, naphthol-AS-BI-phosphohydrolase and α-glucosidase. Negative for Voges–Proskauer test, reduction of nitrates to nitrites, fermentation of glucose, urease, arginine dihydrolase, lysine decarboxylase, ornithine decarboxylase, tryptophan deaminase, α-galactosidase, β-galactosidase, β-glucuronidase, β-glucosidase, *N*-acetyl-β-glucosaminidase, α-mannosidase and α-fucosidase. Utilize the following carbohydrates as the sole carbon source: d-glucose, d-galactose, maltose, lactose, d-mannose, glycogen, l-proline, d-raffinose, sucrose, d-trehalose, cellobiose and l-arabinose. The utilization of melibiose, d-turanose and d-fructose is variable. Under the tested conditions, it does not utilize the following carbohydrates: d-mannitol, d-xylose, l-rhamnose, propionate, tartrate, l-sorbose, citrate, myo-inositol, succinate and d-ribose. The major fatty acids are iso-C_15:0_, anteiso-C_15:0_, iso-C_15:0_ 3-OH and summed feature 3 (C_16:1_* ω*7*c* and/or C_16:1_* *ω*6c*). The genomic DNA G+C content of the type strain is 33.8 mol%.

The type strain XS1P32^T^ (=CGMCC 1.23370^T^=NBRC 115055^T^) was isolated from a meltwater sample collected from the Zhuxi Glacier on the Tibetan Plateau, PR China. The NCBI accession numbers for the 16S rRNA gene and genome sequences are PX401551 and JBLVPT000000000, respectively.

## Description of *Flavobacterium glycogeni* sp. nov.

*Flavobacterium glycogeni* (gly.co.ge’ni. N.L. gen. n. *glycogeni*, of glycogen).

Cells are Gram-stain-negative, rod-shaped, exhibiting gliding motility and devoid of flagella, measuring 0.8–1.0×1.8–3.9 µm. Colonies are circular, convex and orange on PYG plates at 14 °C. Growth occurs at temperatures between 0 and 25 °C (optimum 14–20 °C), at pH 6.0–8.0 (optimum pH 7.0) and in the presence of 0–1.0% (w/v) NaCl. Flexirubin-type pigments are present. Positive for catalase and oxidase. Cells hydrolyse starch, aesculin and gelatin, but do not hydrolyse casein or Tween 80. Indole and H_2_S are not formed. Positive for the Voges–Proskauer test, alkaline phosphatase, esterase (C4), esterase lipase (C8), lipase (C14), leucine arylamidase, valine arylamidase, cystine arylamidase, trypsin, α-chymotrypsin, acid phosphatase, naphthol-AS-BI-phosphohydrolase, α-galactosidase, β-galactosidase, α-glucosidase and *N*-acetyl-β-glucosaminidase. Negative for reduction of nitrates to nitrites, fermentation of glucose, urease, arginine dihydrolase, lysine decarboxylase, ornithine decarboxylase, tryptophan deaminase, β-glucuronidase, β-glucosidase, α-mannosidase and α-fucosidase. Utilize the following carbohydrates as the sole carbon source: d-glucose, d-galactose, melibiose, maltose, lactose, d-mannose, glycogen, l-proline and d-trehalose. Under the tested conditions, it does not utilize the following carbohydrates: d-mannitol, d-xylose, d-turanose, d-fructose, l-rhamnose, propionate, tartrate, l-sorbose, citrate, myo-inositol, succinate, d-ribose, d-raffinose, sucrose, cellobiose and l-arabinose. The major fatty acids are anteiso-C_15:0_, iso-C_15:0_ and summed feature 3 (C_16:1_* ω*7*c* and/or C_16:1_* *ω*6c*). The genomic DNA G+C content of the type strain is 34.5 mol%.

The type strain ZB4P13^T^ (=CGMCC 1.24050^T^=NBRC 115056^T^) was isolated from an ice sample collected from the Zepu Glacier on the Tibetan Plateau, PR China. The NCBI accession numbers for the 16S rRNA gene and genome sequences are PX401553 and JBLVPV000000000, respectively.

## Description of *Flavobacterium kochi* sp. nov.

*Flavobacterium kochi* (ko’chi. N.L. gen. masc. n. *kochi*, of Koch, honouring the German microbiologist Robert Koch).

Cells are Gram-stain-negative, rod-shaped, non-gliding and devoid of flagella, measuring 0.7–0.8×2.4–3.8 µm. Colonies are circular, convex and yellow on PYG plates at 14 °C. Growth occurs at temperatures between 0 and 25 °C (optimum 14–20 °C), at pH 6.0–8.0 (optimum pH 7.0) and in the presence of 0–0.5% (w/v) NaCl. Flexirubin-type pigments are present. Positive for catalase and negative for oxidase. Cells hydrolyse aesculin and gelatin, but do not hydrolyse starch, casein or Tween 80. Indole and H_2_S are not formed. Positive for the Voges–Proskauer test, alkaline phosphatase, esterase (C4), esterase lipase (C8), lipase (C14), leucine arylamidase, valine arylamidase, cystine arylamidase, acid phosphatase, naphthol-AS-BI-phosphohydrolase, α-galactosidase, β-galactosidase, α-glucosidase, β-glucosidase and *N*-acetyl-β-glucosaminidase. Negative for reduction of nitrates to nitrites, fermentation of glucose, urease, arginine dihydrolase, lysine decarboxylase, ornithine decarboxylase, tryptophan deaminase, trypsin, α-chymotrypsin, β-glucuronidase, α-mannosidase and α-fucosidase. Utilize the following carbohydrates as the sole carbon source: d-glucose, d-galactose, melibiose, maltose, lactose, d-mannose, d-xylose, d-turanose, d-fructose, glycogen, l-proline, d-raffinose, sucrose, d-trehalose, cellobiose and l-arabinose. Under the tested conditions, it does not utilize the following carbohydrates: d-mannitol, l-rhamnose, propionate, tartrate, l-sorbose, citrate, myo-inositol, succinate and d-ribose. The major fatty acids are iso-C_15:0_, anteiso-C_15:0_ and summed feature 3 (C_16:1_* ω*7*c* and/or C_16:1_* *ω*6c*). The genomic DNA G+C content of the type strain is 35.3 mol%.

The type strain ZS1P14^T^ (=CGMCC 1.24093^T^=NBRC 114828^T^) was isolated from a meltwater sample collected from the Zepu Glacier on the Tibetan Plateau, PR China. The NCBI accession numbers for the 16S rRNA gene and genome sequences are PX401554 and JBLVPW000000000, respectively.

## Description of *Flavobacterium cryophilum* sp. nov.

*Flavobacterium cryophilum* (cry.o'phi.lum. Gr. neut. n. *kryos*, cold; Gr. masc. adj. *philos*, loving; N.L. neut. adj. *cryophilum*, cold-loving).

Cells are Gram-stain-negative, rod-shaped, non-gliding and devoid of flagella, measuring 0.7–0.8×1.5–4.5 µm. Colonies are circular, convex and yellow on PYG plates at 14 °C. Growth occurs at temperatures between 0 and 21 °C (optimum 14 °C), at pH 6.0–8.0 (optimum pH 7.0) and in the presence of 0–0.5% (w/v) NaCl. Flexirubin-type pigments are absent. Positive for catalase and oxidase. Cells hydrolyse casein and aesculin, but do not hydrolyse gelatin, starch or Tween 80. Indole and H_2_S are not formed. Positive for the Voges–Proskauer test, alkaline phosphatase, esterase (C4), esterase lipase (C8), lipase (C14), leucine arylamidase, valine arylamidase, cystine arylamidase, α-chymotrypsin, acid phosphatase, naphthol-AS-BI-phosphohydrolase, β-galactosidase, α-glucosidase, β-glucosidase and *N*-acetyl-β-glucosaminidase. Negative for reduction of nitrates to nitrites, fermentation of glucose, urease, arginine dihydrolase, lysine decarboxylase, ornithine decarboxylase, tryptophan deaminase, trypsin, α-galactosidase, β-glucuronidase, α-mannosidase and α-fucosidase. Utilize the following carbohydrates as the sole carbon source: d-glucose, d-galactose, maltose, lactose, d-mannose, d-turanose, glycogen, l-proline, d-raffinose, sucrose, d-trehalose, cellobiose and l-arabinose. The utilization of d-fructose is variable. Under the tested conditions, it does not utilize the following carbohydrates: melibiose, d-mannitol, d-xylose, l-rhamnose, propionate, tartrate, l-sorbose, citrate, myo-inositol, succinate and d-ribose. The major fatty acids are iso-C_15:0_ and summed feature 3 (C_16:1_* ω*7*c* and/or C_16:1_* *ω*6c*). The genomic DNA G+C content of the type strain is 34.5 mol%.

The type strain GT2P42^T^ (=CGMCC 1.24821^T^=NBRC 114831^T^) was isolated from a cryoconite sample collected from the Gawalong Glacier on the Tibetan Plateau, PR China. The NCBI accession numbers for the 16S rRNA gene and genome sequences are PX401557 and JBLVPZ000000000, respectively.

## Supplementary material

10.1099/ijsem.0.007021 Uncited Supplementary Material 1.

## Abbreviations

ANI: average nucleotide identity
CDSs: coding sequences
dDDH: digital DNA–DNA hybridization
ML: maximum-likelihood
NJ: neighbour-joining
PYG: peptone, yeast extract and glucose
R2A: Reasoner’s 2A agar
TYGS: Type (Strain) Genome Server
