# Supplementary material for: Ten novel psychrophilic Flavobacterium species from Tibetan Plateau glaciers define a cryospheric lineage with global cold-origin relatives
Source: Int J Syst Evol Microbiol. 2026 Jan 13;76(1):007021. doi: 10.1099/ijsem.0.007021 (PMC12799291; doi:10.1099/ijsem.0.007021)
Supplement: Uncited Supplementary Material 1. [file ijsem-76-07021-s001.pdf]

Supplementary materials

Ten novel psychrophilic *Flavobacterium* species from Tibetan Plateau glaciers define a cryospheric lineage with global cold-origin relatives

Liu Q, Yang LL, Xin YH.

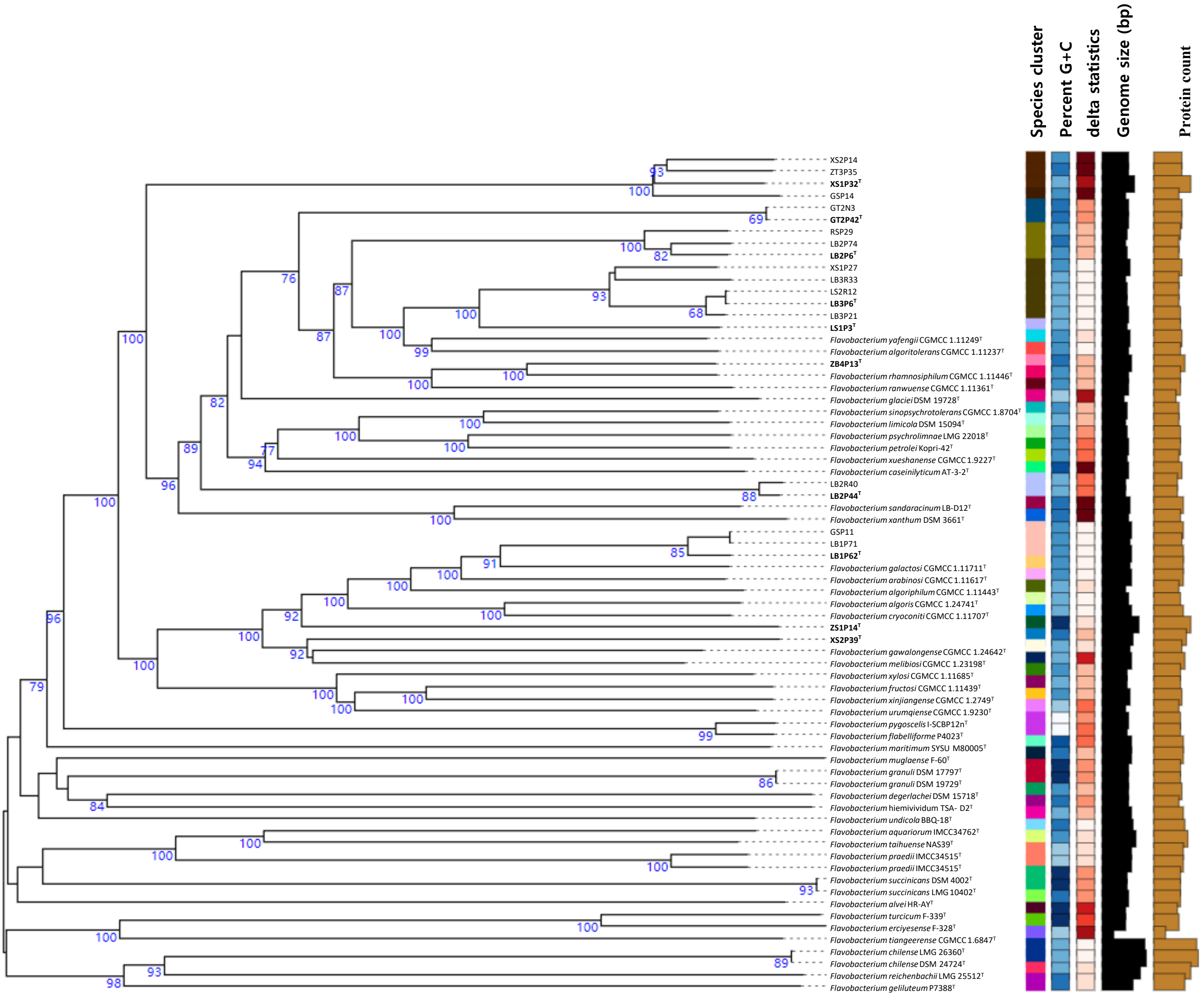

Fig. S1. Genome BLAST distance phylogeny (GBDP) of selected genomes, inferred using the TYGS webserver. Node values are based on 100 pseudo-bootstrap replicates.

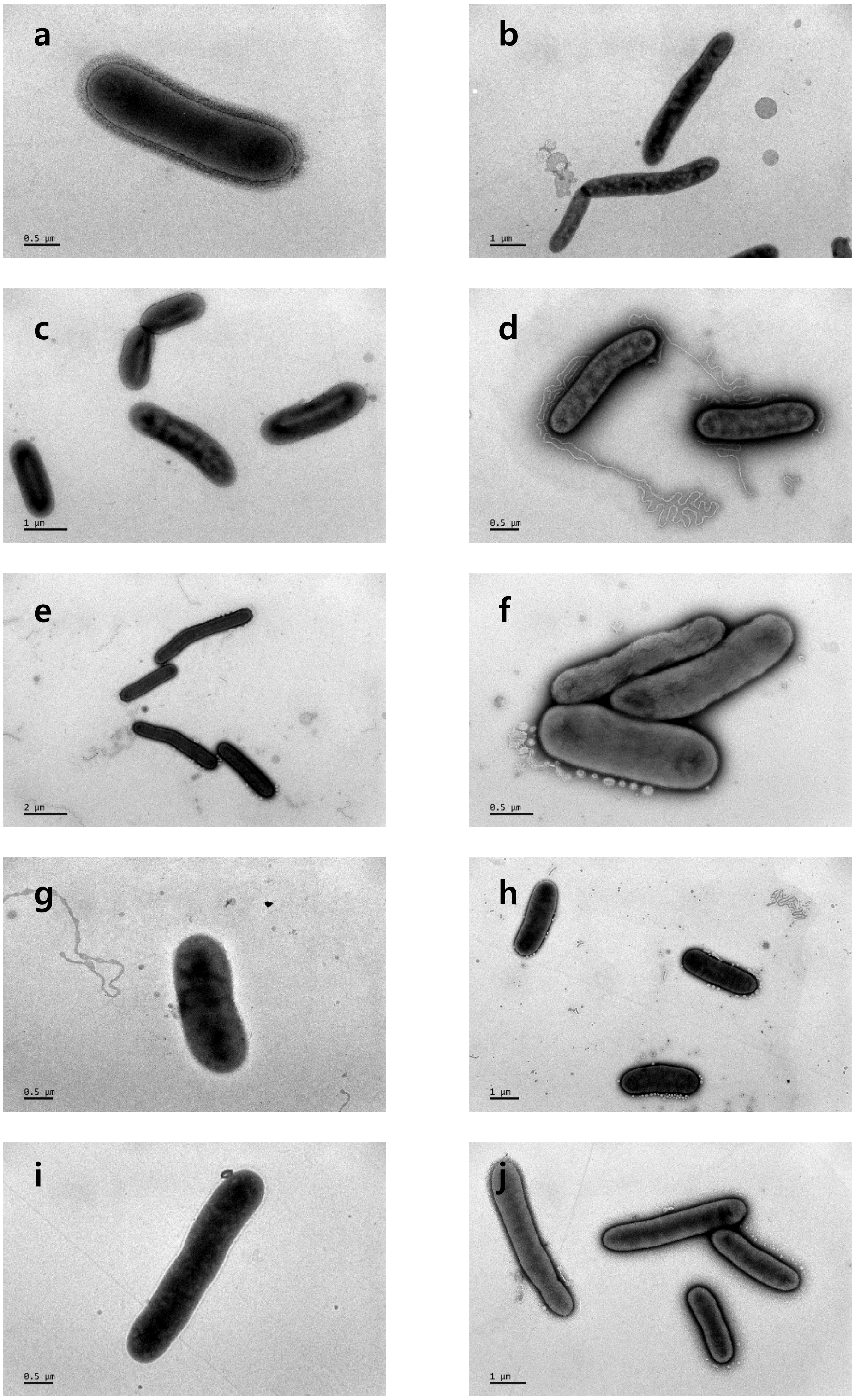

Fig. S2. Transmission electron micrograph of negatively stained cells of strains LB2P44^T (a), LB2P6^T (b), LB1P62^T (c), LB3P6^T (d), LS1P3^T (e), XS2P39^T (f), XS1P32^T (g), ZB4P13^T (h), ZS1P14^T (i), and GT2P42^T (j) grown at 14℃ on PYG agar. All scale bars are shown as solid black horizontal lines. Different magnifications are used across the images, resulting in scale bar lengths of 0.5 μm, 1 μm, or 2 μm as labeled individually in each panel.

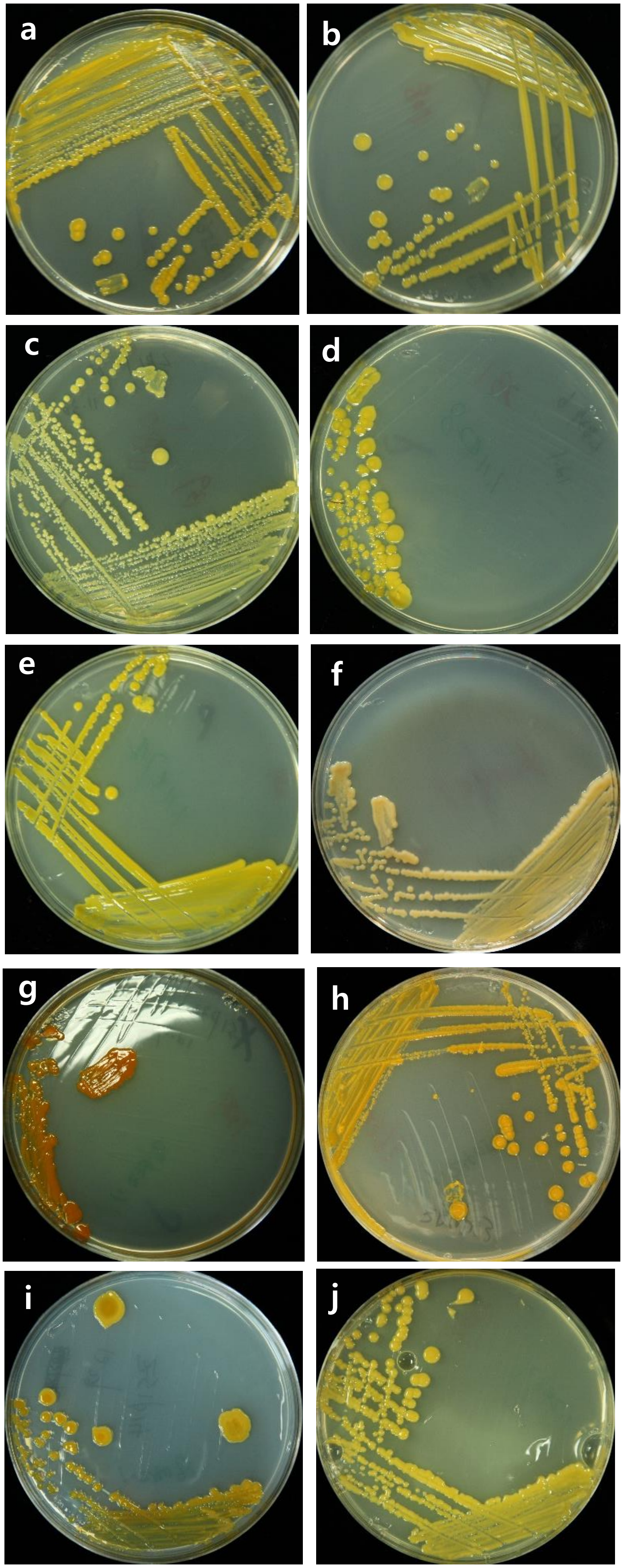

Fig. S3. The colonies of strains LB2P44^T (a), LB2P6^T (b), LB1P62^T (c), LB3P6^T (d), LS1P3^T (e), XS2P39^T (f), XS1P32^T (g), ZB4P13^T (h), ZS1P14^T (i), and GT2P42^T (j) grown at 14℃ on PYG agar.

Table S1. The basic information and the blast result using 16S rRNA gene sequences of the 23 strains.

Strains	16S rRNA gene Acceesion No.	Closest relatives	Similarity (%)
LB2P44^T	PX401538	*Flavobacterium sinopsychrotolerans* 0533^T	98.77
LB2R40	PX401547	*Flavobacterium sinopsychrotolerans* 0533^T	98.74
LB2P6^T	PX401539	*Flavobacterium urumqiense* Sr25^T	98.12
LB2P74	PX401542	*Flavobacterium urumqiense* Sr25^T	98.76
RSP29	PX401556	*Flavobacterium sinopsychrotolerans* 0533^T	98.74
LB1P62^T	PX401540	*Flavobacterium galactosi* ZT3R25^T	99.34
LB1P71	PX401541	*Flavobacterium galactosi* ZT3R25^T	99.49
GSP11	PX401555	*Flavobacterium galactosi* ZT3R25^T	99.56
LB3P6^T	PX401543	*Flavobacterium glaciei* 0499^T	99.04
LB3P21	PX401544	*Flavobacterium glaciei* 0499^T	98.91
LB3R33	PX401546	*Flavobacterium glaciei* 0499^T	99.06
LS2R12	PX401548	*Flavobacterium glaciei* 0499^T	98.41
XS1P27	PX401549	*Flavobacterium glaciei* 0499^T	98.92
LS1P3^T	PX401545	*Flavobacterium algoritolerans* LB1P51^T	98.97
XS2P39^T	PX401550	*Flavobacterium melibiosi* XS2P12^T	98.68
XS1P32^T	PX401551	*Flavobacterium glaciei* 0499^T	98.78
ZT3P35	PX401552	*Flavobacterium glaciei* 0499^T	98.48
GSP14	PX401559	*Flavobacterium glaciei* 0499^T	98.26
XS2P14	PX401560	*Flavobacterium glaciei* 0499^T	98.49
ZB4P13^T	PX401553	*Flavobacterium sinopsychrotolerans* 0533^T	98.69
ZS1P14^T	PX401554	*Flavobacterium arabinosi* LT1R49^T	99.48
GT2P42^T	PX401557	*Flavobacterium melibiosi* XS2P12^T	98.48
GT2N3	PX401558	*Flavobacterium melibiosi* XS2P12^T	98.56

Table S2. Basic genome information for the 23 strains isolated in this study.

Strains	Completeness (%)	Contamination (%)	Contigs	Total length (Mb)	GC (%)	N50	N75	Genome Acceccsion No.
LB2P44^{T}	99.87	0.21	26	3.30	33.94	1079259	1079259	GCA_048284335.1
LB2R40	99.98	0.26	25	3.35	33.91	1129195	1129195	GCA_048286475.1
LB2P6^{T}	99.99	0	38	3.52	34.32	183682	138704	GCA_048284345.1
LB2P74	99.98	0.25	34	3.38	34.41	538737	349621	GCA_048286535.1
RSP29	99.99	0.34	114	3.66	34.27	93644	38214	GCA_048286655.1
LB1P62^{T}	100	0.12	78	4.09	34.17	178824	126734	GCA_048284325.1
LB1P71	99.96	0.26	68	4.07	34.22	250113	106155	GCA_048284385.1
GSP11	99.94	0.1	69	4.17	34.24	237508	94159	GCA_048286595.1
LB3P6^{T}	99.97	0.15	40	3.55	33.85	535749	211946	GCA_048286755.1
LB3P21	99.97	0.5	37	3.70	33.92	584099	459318	GCA_048286835.1
LB3R33	99.96	0.16	54	3.46	33.9	223716	170891	GCA_048286495.1
LS2R12	100	0.17	50	3.56	33.85	332873	211946	GCA_048286815.1
XS1P27	100	0.3	182	3.91	34.08	406206	292827	GCA_048286575.1
LS1P3^{T}	99.95	0.18	36	3.53	33.73	352489	255118	GCA_048286615.1
XS2P39^{T}	100	0.27	88	4.46	34.74	156720	89445	GCA_048286515.1
XS1P32^{T}	100	0.13	44	3.65	33.87	201483	107291	GCA_048286795.2
ZT3P35	100	0.87	236	3.78	34.68	113329	68494	GCA_048286735.1
GSP14	100	0.43	53	3.44	34.04	180611	137912	GCA_048286675.1
XS2P14	100	0.76	100	3.80	34.16	128863	70184	GCA_048286715.1
ZB4P13^{T}	100	0.6	119	4.23	34.47	152076	74340	GCA_048286775.1
ZS1P14^{T}	100	0.98	97	5.23	35.29	174444	84712	GCA_048286695.1
GT2P42^{T}	99.97	0.01	30	3.83	34.51	234644	213819	GCA_048286555.1
GT2N3	99.95	0.24	49	3.86	34.54	230764	183200	GCA_048286635.1

Table S3. Basic information on genome annotation for the 23 strains.

Strain	CDS	Gene	misc RNA	rRNA	Repeat region	tRNA	tmRNA
LB2P44^T	2874	3000	77	5	0	43	1
LB2R40	2915	3038	74	5	0	43	1
LB2P6^T	3102	3275	116	7	0	49	1
LB2P74	2970	3139	115	7	0	46	1
RSP29	3228	3442	158	9	1	46	1
LB1P62^T	3440	3615	119	9	0	46	1
LB1P71	3427	3599	119	7	0	45	1
GSP11	3497	3669	119	8	0	44	1
LB3P6^T	3052	3195	89	7	0	46	1
LB3P21	3198	3349	94	9	0	47	1
LB3R33	3008	3156	93	9	1	45	1
LS2R12	3058	3203	89	8	0	47	1
XS1P27	3368	3518	95	8	0	46	1
LS1P3^T	3115	3256	87	6	1	47	1
XS2P39^T	3793	3932	88	5	1	45	1
XS1P32^T	3181	3347	121	4	0	10	1
ZT3P35	3235	3409	121	5	1	47	1
GSP14	3015	3191	119	8	0	48	1
XS2P14	3325	3508	125	7	0	50	1
ZB4P13^T	3675	3809	81	8	0	44	1
ZS1P14^T	4389	4519	82	5	1	42	1
GT2P42^T	3354	3511	103	9	0	44	1
GT2N3	3348	3504	103	8	0	44	1

Table S4. Carbon source utilization of the six *Flavobacterium* strains in this study.

Strains: 1, LB2P44^T, LB2R40; 2, LB2P6^T, LB2P74, RSP29; 3, LB1P62^T, LB1P71, GSP11; 4, LB3P6^T, LB3P21, LB3R33, LS2R12, XS1P27; 5, LS1P3^T; 6, XS2P39^T; 7, XS1P32^T, ZT3P35, GSP14, XS2P14; 8, ZB4P13^T; 9, ZS1P14^T; 10, GT2P42^T, GT2N3.

**Carbon soures:**	**1**	**2**	**3**	**4**	**5**	**6**	**7**	**8**	**9**	**10**
D-glucose	+	+	+	+	+	+	+	+	+	+
D-galactose	+	+	+	+	+	+	+	+	+	+
melibiose	+	+/-	+	+	+	+	+/-	+	+	-
maltose	+	+	+	+	+	+	+	+	+	+
lactose	+	+	+	+	+	+	+	+	+	+
D-mannitol	-	-	-	-	-	-	-	-	-	-
D-mannose	+	+	+	+	+	+	+	+	+	+
D-xylose	-	-	+	-	-	-	-	-	+	-
D-turanose	+	+	+	+	+	+	+/-	-	+	+
D-fructose	+	+	+	+	+	+	+/-	-	+	+/-
glycogen	+	+	+	+	+	+	+	+	+	+
L-rhamnose	+/-	-	+	-	-	+	-	-	-	-
propionate	-	-	-	-	-	-	-	-	-	-
tartrate	-	-	-	-	-	-	-	-	-	-
L-sorbose	-	-	-	-	-	-	-	-	-	-
citrate	-	-	-	-	-	-	-	-	-	-
myo-inositol	-	-	-	-	-	-	-	-	-	-
succinate	-	-	-	-	-	-	-	-	-	-
D-ribose	-	-	-	-	-	-	-	-	-	-
L-proline	+	+	+	+	-	+	+	+	+	+
D-raffinose	+	+	+	+	+	+	+	-	+	+
sucrose	+	+	+	+	+	+	+	-	+	+
D-trehalose	+	+	+	+	+	+	+	+	+	+
cellobiose	+	+	+	+	+	+	+	-	+	+
L-arabinose	+	+	+	+	+	+	+	-	+	+

Table S5. Cellular fatty acid compositions (%) of strains LB3P122^T, LT1R49^T, ZT3R17^T, ZT3R25^T, XS2P12^T, GB2R13^T, and other related strains.

Strains: 1, LB2P44^T; 2, LB2P6^T; 3, LB1P62^T; 4, LB3P6^T; 5, LS1P3^T; 6, XS2P39^T; 7, XS1P32^T; 8, ZB4P13^T; 9, ZS1P14^T; 10, GT2P42^T. Values are percentages of the total fatty acids. tr, traces (less than 1% of the total fatty acids); -, not detected. *Summed Features are fatty acids that cannot be resolved reliably from another fatty acid using the chromatographic conditions chosen. The MIDI system groups these fatty acids together as one feature with a single percentage of the total. Summed features consist of: 2, *iso*-$C_{16:1}$ I/ $C_{14:0}$ 3-OH; 3, $C_{16:1}$ $\omega 7c$/$C_{16:1}$ $\omega 6c$; 4, $C_{17:1}$ iso I/anteiso $C_{17:1}$ B; 9, iso-$C_{17:1}$ $\omega 9c$/10-methyl $C_{16:0}$.

Fatty acid	1	2	3	4	5	6	7	8	9	10
**Saturated**										
$C_{16:0}$	2.1	2.1	2.3	1.5	1.8	2.7	tr	1.7	2.7	3.4
**Branched**										
iso-$C_{12:0}$	1.9	2.1	-	-	-	-	-	-	-	-
iso-$C_{13:0}$	4.5	2.9	tr	tr	tr	tr	-	1.9	2.2	-
iso-$C_{14:0}$	4.8	3.1	-	tr	-	-	5.1	3.4	-	-
iso-$C_{15:0}$	10.8	12.4	10.9	10.1	8.1	11.5	10.5	10.8	16.8	15.6
anteiso-$C_{15:0}$	3.1	6.5	12.2	5.9	6.8	14.3	10.2	13.1	13.3	8.6
iso-$C_{15:1}$ G	8.1	7.7	5.3	7.5	4.4	5.3	7.2	6.5	5.1	8.1
anteiso-$C_{15:1}$ A	tr	1.0	tr	tr	tr	tr	2.1	1.5	1.0	1.3
iso-$C_{16:0}$	3.1	1.5	1.6	2.1	tr	1.3	2.4	2.6	1.4	6.3
anteiso-$C_{17:1}\omega 9c$	-	-	-	-	-	1.6	-	-	-	-
**Unsaturated**										
$C_{15:1}\omega 6c$	7.0	4.6	5.6	5.9	1.9	5.7	5.6	3.6	4.6	2.4
iso-$C_{16:1}$-H	2.8	0.9	tr	1.5	-	1.2	2.4	2.0	1.0	3.9
$C_{17:1}\omega 6c$	7.0	7.6	7.8	10.7	2.5	6.2	6.5	4.4	7.5	4.0
$C_{17:1}\omega 8c$	tr	1.1	tr	3.1	tr	tr	tr	1.0	1.2	tr
**Hydroxy**										
$C_{15:0}$-3OH	1.5	2.0	-	1.9	1.3	-	2.4	1.7	-	-
iso-$C_{15:0}$-3OH	7.9	11.2	6.5	9.0	16.0	5.3	10.9	9.2	6.2	5.2
iso-$C_{16:0}$-3OH	5.4	4.3	2.3	3.9	1.5	2.2	7.1	3.5	2.3	4.3
$C_{16:0}$-3OH	2.5	2.6	2.0	1.4	4.4	1.2	2.0	1.5	1.7	1.6
iso-$C_{17:0}$-3OH	5.6	7.3	8.1	7.3	8.8	5.9	3.9	8.1	5.2	3.6
**SummedFeature***										
Summed Feature 2	-	tr	tr	-	2.0	tr	tr	tr	tr	tr
Summed Feature 3	12.3	11.6	20.4	13.6	27.6	20.9	11.1	10.1	14.4	18.0
Summed Feature 4	-	-	2.3	-	-	1.1	-	-	2.1	1.4
Summed Feature 9	3.1	-	4.1	8.1	4.5	5.3	2.9	6.1	4.1	4.4
